# Measurement and Geometric Modelling of Human Spine Posture for Medical Rehabilitation Purposes Using a Wearable Monitoring System Based on Inertial Sensors

**DOI:** 10.3390/s17010003

**Published:** 2016-12-22

**Authors:** Gheorghe-Daniel Voinea, Silviu Butnariu, Gheorghe Mogan

**Affiliations:** Department of Automotive and Transport Engineering, Transilvania University of Brasov, 29 Eroilor Blvd, RO-500036 Brasov, Romania; butnariu@unitbv.ro (S.B.); mogan@unitbv.ro (G.M.)

**Keywords:** inertial sensors, wearable system, medical rehabilitation, spine, geometric modelling

## Abstract

This paper presents a mathematical model that can be used to virtually reconstruct the posture of the human spine. By using orientation angles from a wearable monitoring system based on inertial sensors, the model calculates and represents the curvature of the spine. Several hypotheses are taken into consideration to increase the model precision. An estimation of the postures that can be calculated is also presented. A non-invasive solution to identify the human back shape can help reducing the time needed for medical rehabilitation sessions. Moreover, it prevents future problems caused by poor posture.

## 1. Introduction

Spinal disorders are usually caused by incorrect postures adopted during daily activities. Once they appear, medication is the first solution that helps to ease the pain. Then, a special rehabilitation plan, which consists of physical exercises and constant posture control, is undertaken with the purpose of eliminating the spinal disorder. In general, these physical therapies last for long periods and the patient has the tendency to go back the bad posture after a while.

The ongoing technological innovations in the area of sensing systems are creating new and better medical applications for various diseases or spinal disorders. Medical applications are not complete without a software or a method to process the data obtained from the hardware [1]. Physicians and patients need to receive meaningful parameters from the system. This data processing requires rather complex algorithms.

A short summary of the state of the art in spine monitoring systems and spine shape reconstruction is presented in the following paragraphs. The aim of this work is to develop a method to generate the shape of the spine, with data obtained from a wearable monitoring system.

There are several approaches in assessing the shape of the human spine, each with advantages and limitations. In general, the back shape can be reconstructed with the help of mathematical formulas from data obtained from various acquisition systems. The radiological method is commonly used and it implies the segmentation of the image and then measuring the angles [2,3,4]. Recently, 3D models were generated using DICOM images that are obtained from an X-ray computed tomography (or CT scans) [5,6,7,8,9,10,11,12].

Monitoring spine movement with optoelectronic systems can yield very accurate results using a relatively small set of surface markers. However, this type of measurements are constrained to special indoor facilities and usually do not offer information on the rotations of the spine [13,14]. Other methods used by researchers to reconstruct the shape of the spine are based on images that were obtained through invasive [15,16,17] or non-invasive techniques [18,19,20,21].

Portable solutions based on strain-gauge sensors that can be used without supervision have the advantage of monitoring the spinal motion in an unobtrusive manner [22,23]. Usually, an accelerometer is also needed to determine the orientation of the sensor while considering Earth’s gravitational field. The system developed in [24] has the capability of storing measured angular data, at up to 100 Hz. Measurements were taken while participants performed several flexion and extension exercises. To reconstruct the shape of the spine, a 6th order polynomial interpolation was applied. Then, the results were compared with the data from the Vicon [25] optical system, which is considered a gold standard. The authors demonstrated that the SpineDMS system can be used to assess the posture of patients with normal body mass index, but there are accuracy problems in the case of adipose patients. O’Sullivan [26] used the BodyGuard™ strain-gauge based device to monitor lumbo-pelvic sagittal plane movements and found it to be reliable, but not suitable for some postural tasks due to high errors. While most of the orientation devices express motion in degrees, the BodyGuard™ calculates the spinal flexion or extension according to the strain gauge elongation and gives a relative range of motion. The system is able to monitor posture or send real-time postural feedback.

High precision potentiometers are at the base of the CA 6000 Spine Motion Analyzer (CA SMA); a system used for studying the kinematics of the lumbar [27] and cervical [28] spine. However, the CA SMA system is not suitable for measuring the range of motion of the spine.

Another unobtrusive method to evaluate the alignment of the spine is presented in [29]. The aim of the study was to monitor the spine by measuring spring indentation present in a mattress and combine this manual data with an auto generated body shape model.

Inertial sensors have been used to measure spinal motion or the range of motion in many studies. One important aspect in measuring spinal motion with inertial sensors is making the data intuitive and user friendly for the clinicians and patients who will use the system. The data can be transformed into meaningful parameters such as rotation, flexion-extension and lateral bending [30,31]. Theobald [32] measured cervical range of motion with inertial sensors. It was proven that they are a viable and objective method for evaluating spine shapes.

Williams [33] investigated the lumbar spine curvature using fiber-optic sensors. These sensors are able to detect inclination according to the intensity of the transmitted light. A spline curve populated with positional data was used to provide the shape of the spine. The authors found this method appropriate for clinical or home use, with good results in measuring spinal curvature over a longer period. However, it is not a solution for measuring rotations or lateral bending due to the inflexibility of the fiber-optic wire.

A new 3-D mobile measurement system that can assess trunk inclination and spine curvature was developed and evaluated in [34]. The system combines an ultrasound-based device with an inclinometer, which also comes with a personalized data analyzing software.

The novelty of this study consists in the development of a mathematical model of the human spine that can be used in medical rehabilitation sessions. The model can be implemented in a wearable spine monitoring system. It relies on the orientation data from inertial sensors and a known distance between the sensors. A classification of the identified models that correspond to possible spinal disorders is also presented. Thus, by considering the angles measured in different areas of the spine, the mathematical model generates the associated curves of the spine shape. The validation of the model was made offline, without using human subjects. The main advantage for the offline measurement is the predictability of how the system should work, thus allowing us to efficiently evaluate the performance of our system through a statistical analysis.

## 2. Materials and Methods

### 2.1. Measurement System

**Figure 1:**
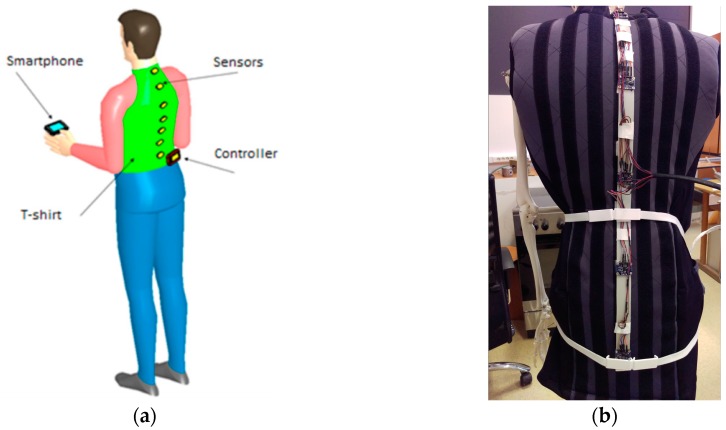
(**a**) Wearable spine monitoring system concept; (**b**) Wearable spine monitoring with flexible straps.

The concept of the spine monitoring system is presented in Figure 1a. It has the following components: a shirt on which the sensors able to detect movement are placed, a controller to collect data from the sensors and transmit it to a smartphone for the user to visualize his/her posture.

**Figure 2:**
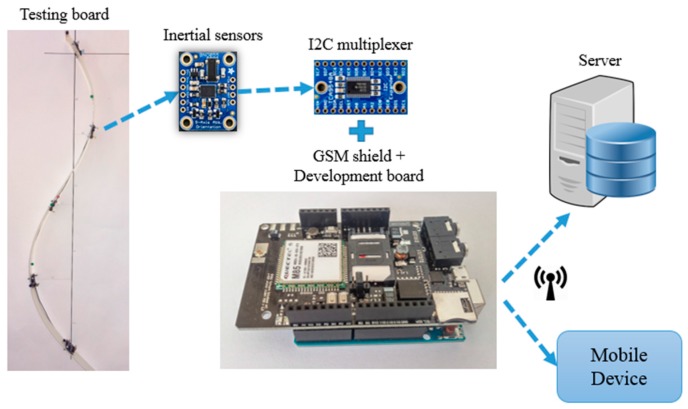
The architecture of the spine monitoring system.

The first prototype uses five sensors (Figure 1b) that are mounted at equal distances, a condition that is needed and sufficient for reconstructing the spine shape with the developed mathematical model. The optimal number of sensors (five) was determined based on the following reasons: the five sensors are distributed evenly on the entire spine in order to detect the curvatures (less than five yield an inaccurate model), the post-processing is not very complex and the system is low cost. The mathematical model calculates and offers meaningful data that can be easily understood by physicians or patients. A spine monitoring system has been developed for acquiring data that describes the posture of the spine. It is based on inertial sensors that are positioned on a flexible frame, which is then fitted on the patient with flexible straps and fabric fasteners. It can communicate wirelessly with a portable device or send the data on a cloud server using GPRS connection. The architecture of the spine monitoring system is presented in Figure 2.

**Figure 3:**
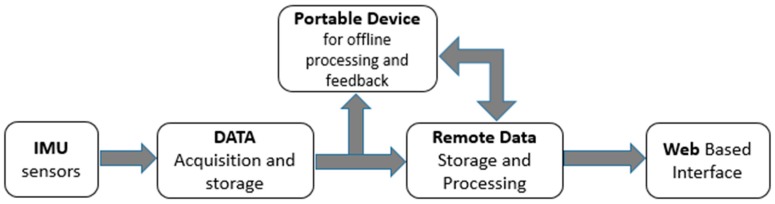
Data flow diagram of the spine monitoring system.

The data that represents the movement of the spine is acquired by the inertial sensors with a development board, stored on a microSD card. The data can be sent periodically to a portable device (smartphone, tablet) or straight to the online server for storage and processing. The Web based interface is used by patients or physicians to access the data, which was already processed and transformed into relevant information (Figure 3).

Inertial sensors are commonly used for monitoring movement. They are also used in smartphones or other small devices, such as fitness bracelets.

### 2.2. Mathematical Model of the Spine

The spine acts as a pillar to support the human body and it protects the spinal cord. When viewed from the side it normally has an S-shape due to three natural curves, which is why the spine can be divided into three regions: cervical, thoracic and lumbar spine.

**Figure 4:**
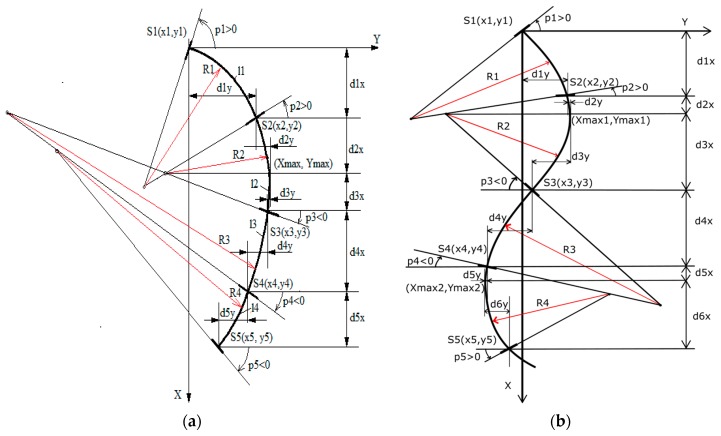
Geometric model: (**a**) C-shape, (**b**) S-shape.

The known data for the mathematical model are five orientation angles (from the inertial sensors) and four distances (between the sensors). We studied three aproaches:

1. A mathematical model based on polynomial functions: a 7th grade polynomial is required that is not time and computational efficient.
2. A mathematical model based on spline functions: cubic functions for each segment. In this case, we ended with a nonlinear sistem of equations that we found to be difficult to use.
3. A mathematical model based on circle arcs (Figure 4): the equations needed to calculate the radius and the coordinates of the imu sensors are only first grade relations. Thus, we get to a linear system of equations that is easier and faster to resolve.

We consider the following hypothesis:

1. The wearable monitoring system provides orientation in 3-axis, but we are only using one axis (using all the axes is the subject of another research article).
2. The proposed mathematical model is based on the approximation of the real curves with circle arcs and geometrical functions.
3. The inputs of the model are the distances measured between the sensors on the flexible frame. The orientation angles are provided by the inertial sensors.
4. The segments formed between two sensors are approximated with arcs of length l1, l2, l3, l4 and radius R1, R2, R3, R4. The circle arcs can have different radius, which in the common point have the same tangent to the slope that is equal with the measured value from the inertial sensors.
5. The circle arcs will be tangent to each other and a smooth curve will be obtained.

Based on the inflection point theory from [35,36], 14 possible models were proposed. These models were classified according to two criteria: the number of maximum points, determined by the coordinates of the point which is furthest from the reference, and the relations between the angles of each inertial sensor. The 14 models are thus grouped into four categories: A—single max point positive curve, B—single max point negative curve, C—double max points positive curve, D—double max negative points curve.

The model can calculate with a very good precision the (X, Y) coordinates of the position of the sensors and reconstruct the natural curves of the spine.

The mathematical model has the following steps (for C-shape), which have been used for all the modelled use cases:

1. Determine the length of the circle arcs generated between two successive sensors: l1, l2, l3 and l4 based on radius (R1, R2, R3, R4) and measured angles (p1, p2, p3, p4, p5);
2. Calculate the distances on X and Y axis for each sensor, except for the first, which is considered a reference: (d1x, d1y), (d2x, d2y), (d3x, d3y), (d4x, d4y), (d5x, d5y);
3. Solve the linear system of 14 equations (Equations (1)–(7) from Table 1) with 14 unknowns (R1, R2, R3, R4, d1x, d1y, d2x, d2y, d3x, d3y, d4x, d4y, d5x, d5y);
4. Calculate the (X, Y) coordinates for each sensor (x2, y2), (x3, y3), (x4, y4) and (x5, y5) and for maximum point (Xmax, Ymax);
5. Calculate the coordinates of the circle center corresponding to the four arcs (see Table 1).

The algorithm for the S-shape is similar with the C-shape algorithm with the difference that it has one more maximum point and the number of equations increases. In the following paragraphs, the equations used for the two scenarios (see Table 2) are presented: a single maximum point curve (a C-shaped curve, which characterizes a kyphosis) and a double maximum point curve (an S-shaped curve, which represents a healthy/normal spine), while the rest are classified according to a set of conditions and presented in Table A1 from the Appendix A. The five sensors of the spine monitoring system create four arcs that will be analytically determined. The distances on the X and Y-axis are calculated if the first sensor is considered as a reference, with the following equations:

The equations above form a system of linear equations that can be solved automatically with a software such as MATLAB or Maple. Equation (1) represent the formulas for the length of an arc. The following six are used to calculate the distances on the X and Y axis of the sensors. Equations (8)–(13) describe the coordinates of the sensors and the maximum points of the curves. The last step is to calculate the coordinates of the center of the circle, for each arc. In order for the prototype to work with the mathematical model the following must be taken into consideration:

- The distance between the inertial sensors must be equal (and known), depending on the length of the spine;
- The position of the first sensor to be at the C7 vertebrae, which in general can be easily palpated;
- The prototype must be in contact with the skin. In the lumbar area of the spine, where there is a more pronounced curve, a flexible strap with a soft material can be used to push the sensing array closer to the spine.

### 2.3. Simulation of Mathematical Model

In order to test the mathematical model for the shape of the spine, the equations presented in Section 2.2 were implemented in Maple [37], a mathematical and analytical software. The models were simulated using possible angles that follow the classification criteria and a distance between sensors of 150 mm. This value was chosen because the average length of the male human spine is about 710 mm and the five sensors are equidistant.

### 2.4. Hardware Equipment Analysis

The main components of the spine shape monitoring system that were tested and presented are the acquisition and control board and the inertial measurement units.

#### 2.4.1. Testing the Acquisition Board

We used the following methodology: identify three acquisition boards that could be used in the spine monitoring system, develop a program to collect data from inertial sensors and analyze the results, taking in consideration the ease of use, technical performance and size.

We reached the following conclusions: the Intel Edison board has an advantage regarding the technical performances, Bluetooth capability and microSD adapter, but the Teensy and Arduino Due are better in terms of price, size, ease of use and energy efficiency.

#### 2.4.2. Testing the Sensors

**Figure 5:**
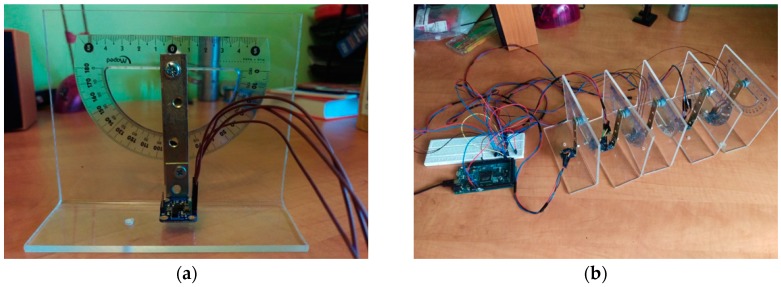
(**a**) Device for testing IMUs; (**b**) Testing equipment for simultaneously testing five IMUs.

A special device was used to test the inertial sensors. One sensor can be tested at a time, by precisely positioning it at a certain predetermined angle (Figure 5a). In order to simultaneously test a larger number of sensors we used a prototyping board and an I2C multiplexer. The test equipment needed to test five inertial sensors is presented in Figure 5b.

Using the same methodology as for the acquisition boards, we identified five different inertial measurement units: Bosch BNO055, MPU 9150, Flora LSM9DS0, AltIMU 10 v.4, MinIMU 9 V3. A comparative analysis is presented in Table 3.

After an analysis of the five IMUs regarding datasheet characteristics, ease of use and data reliability we chose the Bosch BNO055 sensors. These sensors can provide absolute orientation due to the built in sensor fusion algorithms and auto calibration function. The high speed ARM Cortex-M0 processor processes the raw data from the accelerometer, gyroscope and magnetometer and outputs data in the following usable formats: quaternions, Euler angles or vectors. Regarding the supply voltage and supported communication protocols, all tested sensors have similar results.

The next step was to simultaneously test five BNO055 sensors that were connected to a Teensy 3.1 development board, through an I2C multiplexer (Figure 5b).

The Bosch BNO055 (Sety Robotics, Bucharest, Romania) 9-axis absolute orientation sensor is used for the spine monitoring system due to its small size of 20 mm × 27 mm × 4 mm, auto-calibration function, real-time measurement capability and because of the efficient fusion algorithm that is already implemented. Although the sensor is capable of sensing orientation on 3-axis, in this study we focus only on one axis.

Before the spine monitoring system is ready to be used, it needs to be calibrated. There are two steps in calibrating the system: the first step is the IMU calibration: the BNO055 have an auto-calibration function, this requires moving it for a few seconds, while making a circular “∞” pattern. The second step is the patient calibration: the patient is instructed to stay in a specific position and the data captured by the system will serve as a reference. Every update received from the sensors will be compared with this reference values. The patient calibration is a subject for a future research.

#### 2.4.3. Testing the Wireless Communication

We considered two methods of data transmission from the acquisition board: with a Bluetooth adapter that will send the data to a mobile device or with a GSM-GPRS module that will upload the data on the server. The two solutions were tested using the Sparkfun Bluetooth Mate Silver [38] and A-GSM 2.064 module [39].The central component of the A-GSM 2.064 module is the Quectel modem that is capable of connecting to the Internet using TCP, UDP, HTTP or FTP. We chose FTP to upload data to a server using AT commands. Use of a GSM-GPRS module has the advantage of directly connecting the wearable monitoring system to the Internet, but that comes with a smaller autonomy due to the energy that the module needs in order to use GSM or GPRS.

#### 2.4.4. Testing the Local Data Storage

A wearable monitoring system must be capable of storing data throughout the day. The solution is to use a microSD card adapter because of the small size and ease of use. Using Serial Peripheral Interface we connected the card reader to the development board and tested saving the orientation data in a text file, with the values separated with a comma for easier post processing.

### 2.5. Measurement Protocol

**Figure 6:**
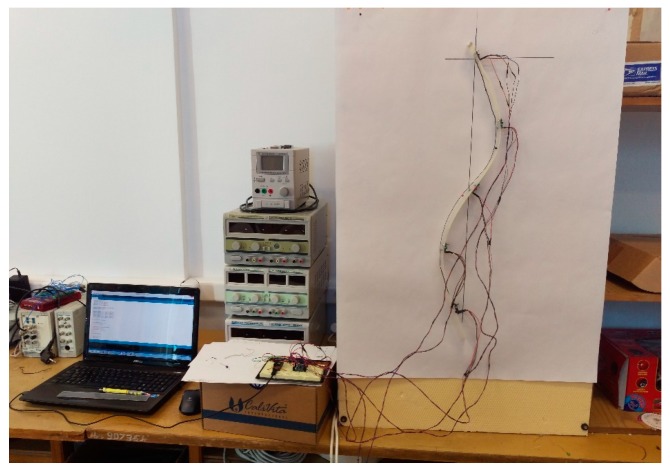
Testing stand.

The testing stand is presented in Figure 6. Measurements are taken in static positions and several use cases were studied and modeled. Calibration is repeated every time the system is disconnected from the power source, although calibration data can be saved and used for future measurements.

**Figure 7:**
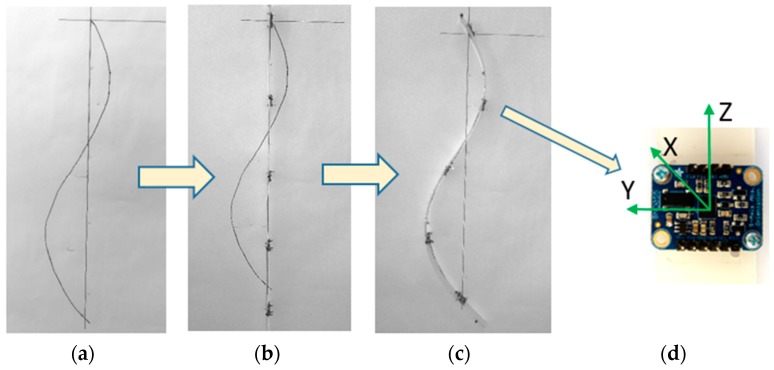
Procedure for testing the mathematical model: (**a**) A theoretical spine shape; (**b**) Getting the reference position; (**c**) The system is fitted to correspond to the plotted shape; (**d**) The inertial sensors collect orientation data.

In Figure 7, the flow of the measurement protocol is presented. A theoretical spine shape is plotted and used on a testing table, and then inertial sensors capture reference values. Lastly, the spine monitoring system is positioned in order to correspond with the drawing. The data from the sensors are saved and are used by the mathematical model to calculate the coordinates of the sensors, using a Maple application. All use cases have been tested using this method.

### 2.6. Testing Methodology and Results

**Figure 8:**
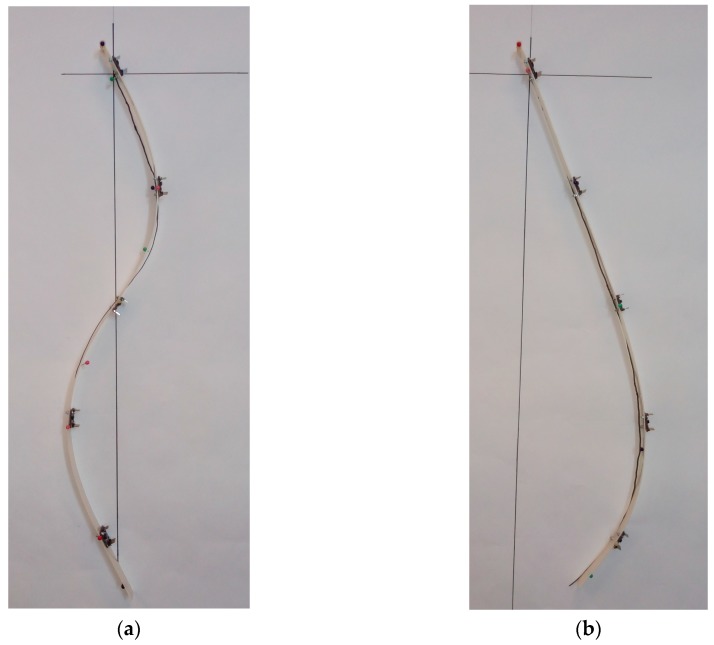
(**a**) C2 posture and (**b**) A4 posture used for measurements.

Two types of posture were chosen for further testing, specifically A4 and C2 (Figure 8). Ten measurements were taken for each shape model (a total of 20 tests), based on the procedure presented in Figure 7. Thus, the flexible spine monitoring system was positioned on top of the plotted shape on a paper and the orientation data was saved. After reading the data from the five sensors, the system was removed and then repositioned after verifying the IMUs calibration status. The repeatability of measurements was taken into consideration, as well as the precision. The saved data from the C2 and A4 postures was then processed using the XLSTAT software [40].

#### 2.6.1. C2 Posture

The quantitative data that resulted is presented in Table 4.

**Figure 9:**
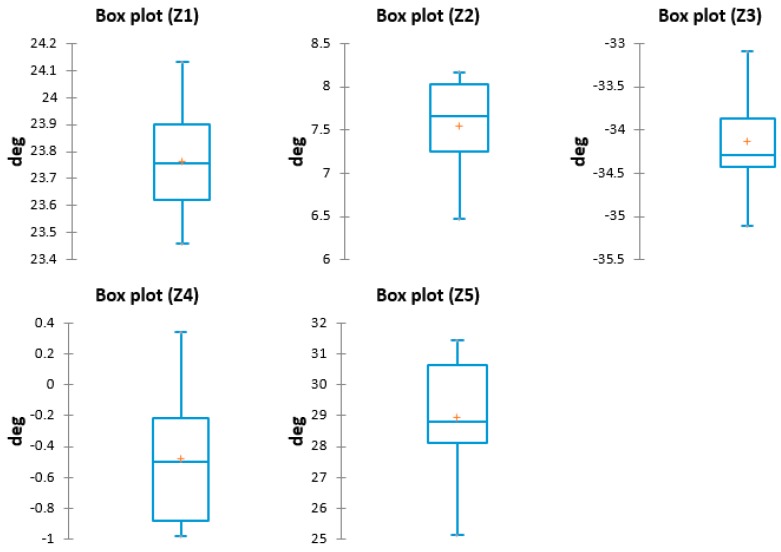
Box plots—C2.

Before analyzing the data using advanced statistical methods, it is customary to check the box chart type (or whisker diagram), which is a simple and complete representation of the obtained results. Thus, we can identify the data trends, anomalies locations and also visualize the minimum, maximum and medium data values. The box plots for the C2 posture are presented in Figure 9.

In statistics, dispersion is a method used to describe how spread out a set of data is. The most common examples of statistical dispersion are standard deviation, variance and interquartile range. Variance represents the arithmetic mean of squared deviations of individual values of a statistical set of experiences, from the arithmetic mean of the whole set. Standard deviation is used to measure the amount of variation or dispersion from the arithmetic mean of a set of data. It is obtained by calculating the square root of variance and it is the most commonly used indicator to characterize a data set, after the average indicator.

#### 2.6.2. A4 Posture

The quantitative data that resulted is presented in Table 5.

**Figure 10:**
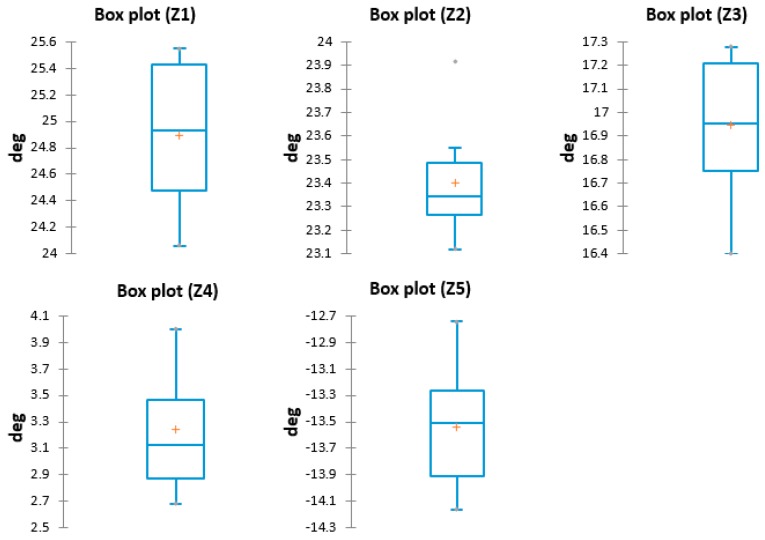
Box plots—A4.

The box plots for A4 posture are presented in Figure 10.

### 2.7. Visual Representation of Obtained Measurements

After an in depth analysis of the measurement data and the statistical results, we determined that there is a cumulative error in the mathematical model that we eliminated by introducing compensation values in the equations (the equations are solved in cascade; due to this fact, the error propagates from the first equation to the last with an increasing effect).

**Figure 11:**
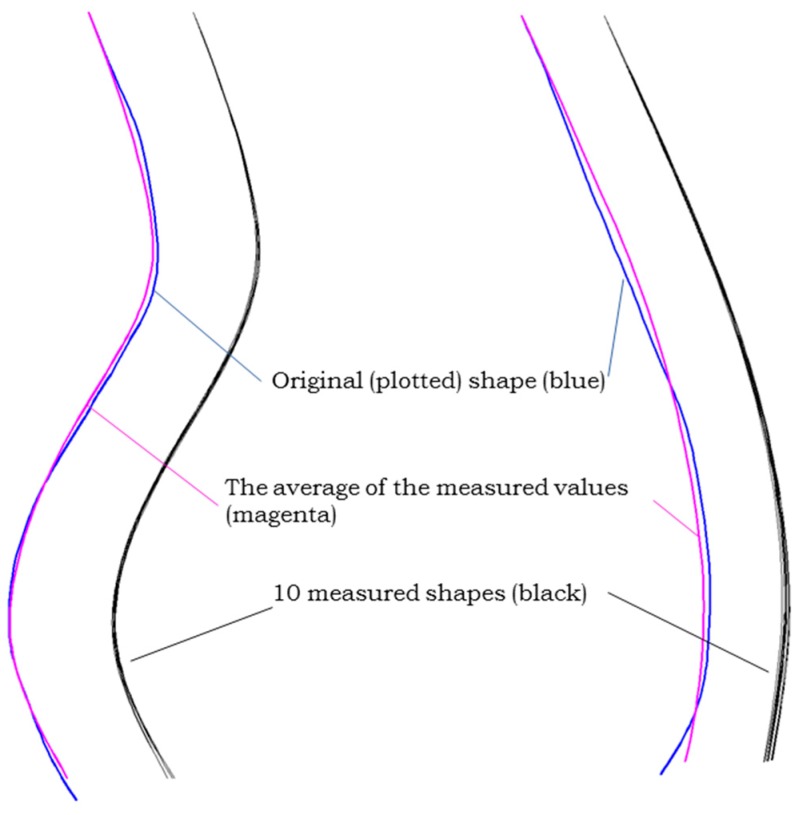
Visual representation of obtained results.

A graphical method was chosen in order to visualize the results of the measurements (Figure 11). Thus, the 10 results for each individual shape model were used with the Maple application we developed. It outputs the coordinates of points where the inertial sensors were positioned, the coordinates of the circle center and corresponding radius. The resulted data was then used with AUTOCAD to draw 10 curves (black color), that are compared with a posture (magenta color) obtained using the average values from the inertial sensors. The resulted shapes were compared with the initial curve plotted on the paper (blue color).

**Figure 12:**
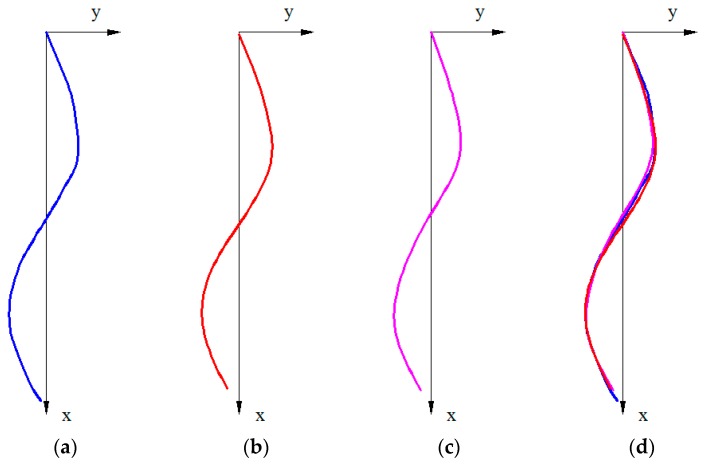
(**a**) Plotted curve; (**b**) Reconstructed curve, using SPLINE command from AutoCAD; (**c**) Reconstructed curve using circle arcs; (**d**) Superimposed curves from (**b**) and (**c**).

**Figure 13:**
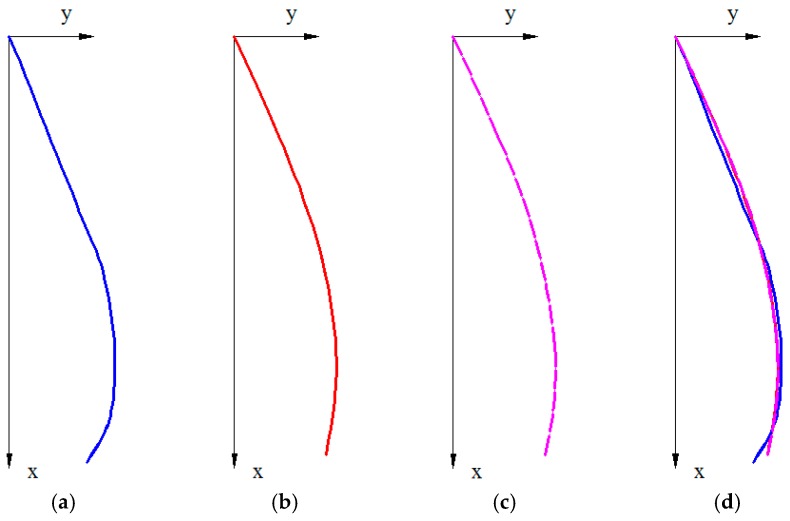
(**a**) Plotted curve; (**b**) Reconstructed curve, using SPLINE command from AutoCAD; (**c**) Reconstructed curve using circle arcs; (**d**) Superimposed curves from (**b**) and (**c**).

The graphical results of the proposed mathematical model was compared using the following method: the coordinates of the points P1 to P5, calculated by the mathematical model using an average of the sensor data, were graphically represented in AUTOCAD; two types of curves were obtained, one using the SPLINE command and the second is based on the arc circle coordinates. The results are presented in Figure 12 for posture C2 and for posture A4 in Figure 13.

#### Error assessment

The procedure for assessing the displacement error of the mathematical model is the following:

1. The spine monitoring system is positioned in a C and S-shape corresponding to models A4 and C2;
2. The orientation angles from the inertial sensors are given to the simulation software in which the mathematical model is implemented;
3. The mathematical model calculates the coordinates of the sensors;
4. The real coordinates of the sensors are manually measured on the testing table;
5. We analyze the results from the simulation software with the manually measured distances.

**Figure 14:**
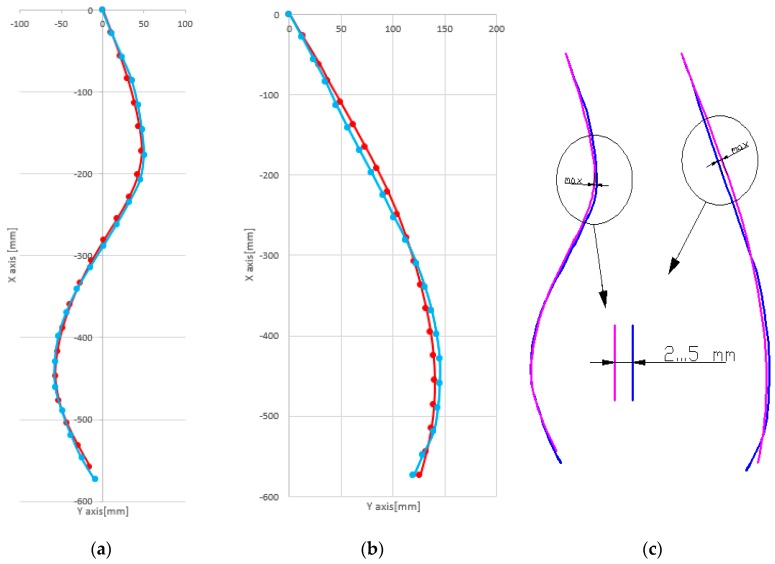
Measured and original posture for C2 posture (**a**) and A4 posture (**b**); Graphical representation of errors (**c**).

The cumulative error from the mathematical model was reduced to less than 5 mm and the reproduced spine shape has a high degree of fidelity (see Figure 14).

Considering that the maximum error percentage is less than 5%, we can conclude that the developed mathematical model can successfully reproduce the spine curvatures and is suitable for use in medical rehabilitation applications or postural monitoring.

## 3. Conclusions

A mathematical model that can be used to reproduce the shape of the spine with data from a wearable monitoring system was presented. The aim of the model is to be used in medical rehabilitation sessions and to help patients to avoid incorrect postures. The simulations presented very good results and demonstrated that the mathematical model can be used to estimate the coordinates of the sensors, which leads to a realistic reconstruction of the spine shape using inertial sensors.

The mathematical model was tested using real orientation angles of the spine monitoring system described in the previous sections. The repeatability of measurements is a key component of precision in any measurement system. The test-retest reliability has been assessed by using two scenarios, one using a C-shape model and one using an S-shape model.

The objective data from the inertial sensors throughout the day also helps physicians to give personalized physical exercises. Based on the inflections point of the spine, 14 possible models were identified and simulated.

The current mathematical model only uses one axis from the inertial sensors, but we plan to expand the model in order to reconstruct movement in all the directions. This advanced model will be used for spinal disorders, such as kyphosis, lordosis and scoliosis. Advances in technology have a direct impact in medical applications and together with software innovations could help to create new and better solutions for various human diseases.

## Figures and Tables

**Table 1:** Equations used by the mathematical model.

Single Max Point Curve (“C” Shape)	Double Max Point Curve (“S” Shape)	Equation Number
l1=R1×(p1−p2) l2=R2×(p2+p3) l3=R3×(p4−p3) l4=R4×(p5−p4)	l1=R1×(p1−p2) l2=R2×(p2+p3) l3=R3×(p3−p4) l4=R4×(p4+p5)	(1)
d1x=R1×(sin(p1)−sin(p2)) d1y=R1×(cos(p2)−cos(p1))	d1x=R1×(sin(p1)−sin(p2)) d1y=R1×(cos(p2)−cos(p1))	(2)
d2x=R2×sin(p2) d2y=R2×(1−cos(p2))	d2x=R2×sin(p2) d2y=R2×(1−cos(p2))	(3)
d3x=R2×sin(p3) d3y=R2×(1−cos(p3))	d3x=R2×sin(p3) d3y=R2×(1−cos(p3))	(4)
d4x=R3×(sin(p4)−sin(p3)) d4y=R3×(cos(p3)−cos(p4))	d4x=R3×(sin(p3)−sin(p4)) d4y=R3×(cos(p4)−cos(p3))	(5)
d5x=R4×(sin(p5)−sin(p4)) d5y=R4×(cos(p4)−cos(p5))	d5x=R4×(sin(p4)) d5y=R4×(1−cos(p4))	(6)
	d6x=R4×(sin(p5)) d6y=R4×(1−cos(p5))	(7)
x2=d1x y2=d1y	x2=d1x y2=d1y	(8)
xmax=x2+d2x ymax=y2+d2y	xmax1=x2+d2x ymax1=y2+d2y	(9)
x3=xmax+d3x y3=ymax−d3y	x3=xmax1+d3x y3=ymax1−d3y	(10)
x4=x3+d4x y4=y3−d4y	x4=x3+d4x y4=y3−d4y	(11)
x5=x4+d5x y5=y4−d5y	xmax2=x4+d5x ymax2=y4−d5y	(12)
	x5=xmax2+d6x y5=ymax2+d6y	(13)
xc1=R1×sin(p1) yc1=−(R1×cos(p1))	xc1=R1×sin(p1) yc1=−(R1×cos(p2)−d1y)	(14)
xc2=d1x+R2×sin(p2) yc2=−(R2−d1y−d2y)	xc2=d1x+R2×sin(p2) yc2=−(R2−d1y−d2y)	(15)
xc3=d1x+d2x+d3x−R3×sin(p3) yc3=−(R3×cos(p3)−d1y−d2y+d3y)	xc3=d1x+d2x+d3x+R3×sin(p3) yc3=R3×cos(p3)+d1y+d2y−d3y	(16)
xc4=d1x+d2x+d3x+d4x+d5x−R4×sin(p5) yc4=−(R4×cos(p4)−d1y−d2y+d3y+d4y)	xc4=d1x+d2x+d3x+d4x+R4×sin(p4) yc4=R4+d1y+d2y−d3y−d4y−d5y	(17)

**Table 2:** Estimation of the postures that can be reproduced by the mathematical model.

Name	Criteria	Results
**A4**	Max point between P4 and P5; p1 > p2, p2 > p3, p3 > p4, p4 < p5.	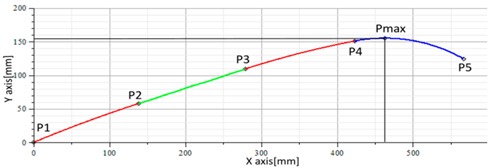
**C2**	Max point between P2-P3 and P4-P5; p1 > p2, p2 < p3, p3 > p4, p4 < p5.	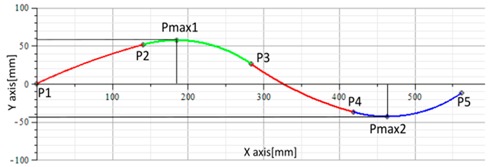

**Table 3:** Inertial sensors comparison.

Characteristic	Bosch BNO055	MPU 9150	Flora LSM9DS0	AltIMU 10 v.4	MinIMU 9 V3
***Accelerometer***					
**Measurement range**	±2 g to ±16 g	±2 g to ±16 g	±2 g to ±16 g	±2 g to ±16 g	±2 g to ±16 g
**Sensitivity**	1 LSB/g	2 LSB/g	0.732 LSB/g	0.732 LSB/g	0.732 LSB/g
***Gyroscope***					
**Measurement range**	±125°/s to ±2000°/s	±250°/s to ±2000°/s	±245°/s to ±2000°/s	±245°/s to ±2000°/s	±245°/s to ±2000°/s
**Sensitivity**	16 LSB/°/s	16.4 LSB/°/s	70 LSB/°/s	70 LSB/°/s	70 LSB/°/s
***Magnetometer***					
**Measurement range**	±1300 μT (x-, y-axis); ±2500 μT (z-axis)	±1200 µT	200 µT to 1200 µT	200 µT to 1200 µT	200 µT to 1200 µT
**Resolution**	0.3 μT	0.3 μT	0.48 μT	0.47 μT	0.47 μT
***Temperature sensor***	Yes	Yes	Yes	No, includes a 24 bits barometer	No
***Size (mm)***	20 × 27 × 4	15.5 × 29 × 4	Diameter of 16 mm, Width 0.8 mm	25.4 × 12.7 × 2.54	20 × 13 × 3
***Communication***	HID-I2C/I2C/UART	I2C	SPI/I2C	I2C	I2C
***Supply voltage***	2.4 V to 3.6 V	2.4 V to 3.46 V	2.4 V to 3.6 V	2.5 V to 5.5 V	2.5 V to 5.5 V
***Power management***	Yes, with three modes	No	Yes	No	No

**Table 4:** Descriptive statistics for posture C2 (quantitative data).

Statistic	Z1	Z2	Z3	Z4	Z5
**Nbr. of observations**	10	10	10	10	10
**Minimum**	23.460	6.470	−35.110	−0.980	25.140
**Maximum**	24.130	8.170	−33.090	0.340	31.440
**1st Quartile**	23.620	7.253	−34.423	−0.878	28.123
**Median**	23.755	7.660	−34.285	−0.500	28.790
**3rd Quartile**	23.900	8.028	−33.865	−0.215	30.638
**Mean**	23.762	7.550	−34.134	−0.477	28.962
**Variance (*n* − 1)**	0.050	0.371	0.369	0.192	3.796
**Standard deviation (*n* − 1)**	0.223	0.609	0.607	0.438	1.948

**Table 5:** Descriptive statistics for posture A4 (quantitative data).

Statistic	Z1	Z2	Z3	Z4	Z5
**Nbr. of observations**	10	10	10	10	10
**Minimum**	24.060	23.120	16.400	2.680	−14.160
**Maximum**	25.550	23.920	17.280	4.000	−12.740
**1st Quartile**	24.475	23.265	16.750	2.870	−13.908
**Median**	24.930	23.345	16.955	3.130	−13.510
**3rd Quartile**	25.430	23.488	17.210	3.463	−13.263
**Mean**	24.889	23.401	16.948	3.238	−13.536
**Variance (*n* − 1)**	0.334	0.05	0.084	0.211	0.188
**Standard deviation (*n* − 1)**	0.578	0.223	0.290	0.460	0.434

## Appendix A

**Table A1:** Estimation of the postures that can be reproduced by the mathematical model.

Name	Criteria	Results
**A1**	Max point between P2 and P3; p1 > p2, p2 < p3, p3 < p4, p4 < p5.	
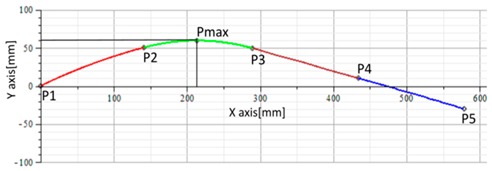
**A2**	Max point between P3 and P4; p1 > p2, p2 > p3, p3 < p4, p4 < p5.	
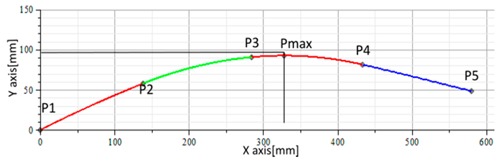
**A3**	Max point between P1 and P2; p1 > p2, p2 < p3, p3 < p4, p4 < p5.	
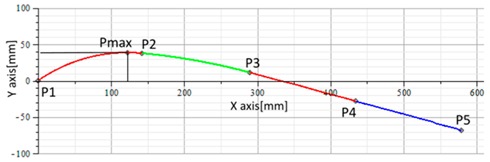
**A4**	Max point between P4 and P5; p1 > p2, p2 > p3, p3 > p4, p4 < p5.	
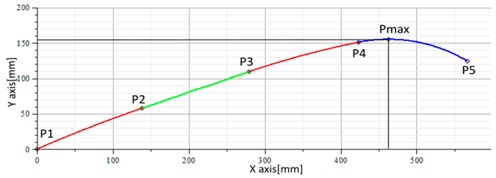
**B1**	Max point between P2 and P3; p1 > p2, p2 < p3, p3 < p4, p4 < p5.	
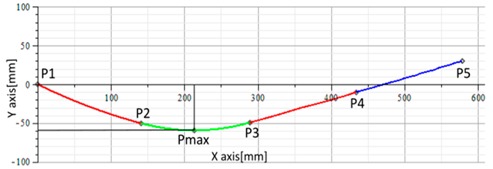
**B2**	Max point between P3 and P4; p1 > p2, p2 > p3, p3 < p4, p4 < p5.	
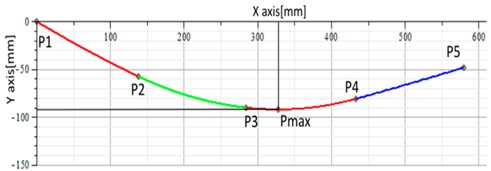
**B3**	Max point between P1 and P2; p1 > p2, p2 < p3, p3 < p4, p4 < p5.	
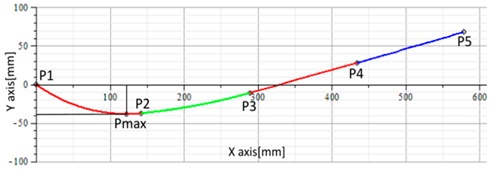
**B4**	Max point between P4 and P5; p1 > p2, p2 > p3, p3 > p4, p4 < p5.	
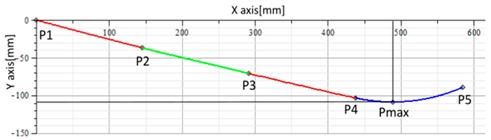
**C1**	Max point between P1-P2 and P3-P4; p1 > p2, p2 < p3, p3 > p4, p4 < p5.	
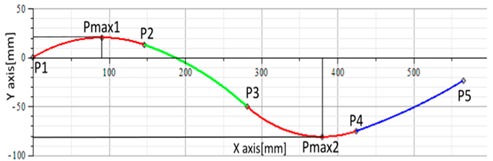
**C2**	Max point between P2-P3 and P4-P5; p1 > p2, p2 < p3, p3 > p4, p4 < p5.	
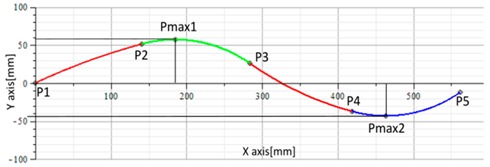
**C3**	Max point between P1-P2 and P3-P4; p1 > p2, p2 < p3, p3 > p4, p4 < p5.	
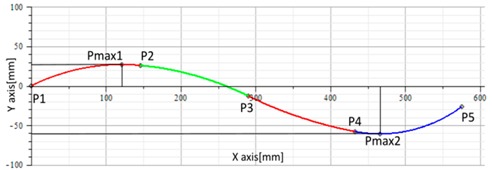
**D1**	Max point between P1-P2 and P3-P4; p1 > p2, p2 < p3, p3 > p4, p4 < p5.	
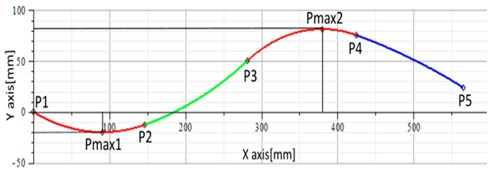
**D2**	Max point between P2-P3 and P4-P5; p1 > p2, p2 < p3, p3 > p4, p4 < p5.	
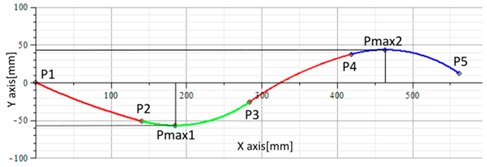
**D3**	Max point between P1-P2 and P3-P4; p1 > p2, p2 < p3, p3 > p4, p4 < p5.	
